# Antigen presenting cells may be able to distinguish between normal and radiated *Schistosoma japonicum* cercaria: an *in vitro* observation^☆^

**DOI:** 10.1016/S1674-8301(10)60040-1

**Published:** 2010-07

**Authors:** Guixia Tang, Minjun Ji, Haiwei Wu, Guanling Wu

**Affiliations:** a Department of Pathogen Biology and Immunology, Nanjing Medical University, Nanjing 210029, Jiangsu Province, China; b Jiangsu Province Key Laboratory of Modern Pathogen Biology, Nanjing 210029, Jiangsu Province, China

**Keywords:** *Schistosoma japonicum*, ultraviolet-radiation-attenuated cercaria, RAW 264.7 cells, normal cercaria, major histocompatibility complex II

## Abstract

**Objective:**

To observe the discrepancies of responses induced by *Schistosoma japonicum* (*S. japonicum*) normal cercaria antigen (NCA) and ultraviolet (UV) -radiation-attenuated cercaria antigen (UVACA) in an *in vitro* system.

**Methods:**

*S. japonicum* cercariae were collected and UVACA and NCA were prepared. Mouse macrophage model cells (RAW 264.7) were treated with medium, NCA (40 µg/mL) or UVACA (40 µg/mL) in the presence or absence of recombinant mouse interferon gamma (rmIFN-γ; 4 ng/mL) for 48 h. Cell surface staining and flow cytometry were used to assess the major histocompatibility complex (MHC)γ; 4 ng/mL) for 48 h. Cell surface staining and flow cytometry were used to assess the major histocompatibility complex (MHC) II expression, and data were expressed as mean fluorescence intensities (MFI). Interleukin (IL) -10, IL-6 and prostaglandin E2 (PGE_2_) in cell culture supernatant were evaluated by commercial enzyme-linked immunosorbent assays.

**Results:**

NCA significantly suppressed IFN-γ-induced MHC II expression on RAW 264.7 cells. In the presence of IFN-γ, NCA significantly promoted IL-6, IL-10 and PGE_2_ secretion from RAW 264.7 cells. In the presence of IFN-γ, UVACA significantly promoted IL-10 but not IL-6 and PGE_2_ secretion from RAW 264.7 cells and showed no effect on IFN-γ-induced MHC II expression. Compared with UVACA, NCA significantly suppressed IFN-γ-induced MHC II expression and significantly promoted IL-6, PGE_2_ and IL-10 secretion from RAW 264.7 cells.

**Conclusion:**

RAW 264.7 cells respond differently to NCA and UVACA. NCA can significantly suppress IFN-γ-induced MHC II expression and significantly promote IL-6, IL-10 and PGE_2_ secretion from RAW 264.7 cells compared with UVACA.

## INTRODUCTION

Schistosomiasis has been recognized as the most important human helminth infection in terms of morbidity and mortality, and it is still a major public health problem in the world[1]. Mass treatment does not prevent re-infection. Furthermore, there is increasing concern over the development of parasite resistance to praziquantel. Consequently, vaccine strategies may represent an essential component for the future control of schistosomiasis as an adjunct to chemotherapy[2]. An improved understanding of the immune response to schistosome infection would facilitate the development of an effective anti-schistosome vaccine. Independent testing of six candidate *Schistosoma mansoni* (*S. mansoni*) antigens: glutathione S-transferase 28 (Sm28-GST), paramyosin, Ir-V5, triose-phosphate isomerase, Sm23, and Sm14, resulted in protective responses being recorded, but the stated goal of a consistent induction of 40% protection or better was not reached with any of the antigens tested[2]. Exposure to radiation-attenuated cercariae remains the best approach for eliciting a consistently high level of protective immunity against schistosome infection. The underlying molecular and immunological mechanisms still need intensive investigation. Comparative soluble proteomic analysis of normal cercariae (NC) and ultraviolet (UV) -radiationattenuated cercariae (UVAC) from *Schistosoma japonicum* (*S. japonicum*) revealed that some proteins showed significant differential expression in the parasite after treatment with ultraviolet light[3]. It also has been proved that the inoculation of radiation-attenuated *S. japonicum* cercaria induce different immune responses from those of a normal cercaria infection[4], both in skin and the local skin-draining lymph nodes (sdLNs) and in spleen. UV-radiation-attenuated *S. japonicum* cercariae elicited predominantly a Th1 response in mice at the early stage of the infection, whereas normal cercariae stimulated primarily Th2-dependent responses[4]. Interferon-γ (IFN-γ) levels were significantly higher in vaccinated mice than in infected mice in the sdLNs in the early stage of the infection[4]. It is well-known that IFN-γ acts as a potent activator of macrophages and plays a pro-inflammatory role, exerting key effects in antagonzing pathogen infection. Interleukin (IL) -12 and IL-4, the potent inducers of Th1 and Th2 responses, respectively, as well as IL-10, showed no differences between the infected and vaccinated mice[4]. These observations imply that IFN-γ might represent a key cytokine in regulating the different immune responses induced by normal and radiated cercaria.

The decision to activate immune responses is made by antigen-presenting cells (APCs) that are quiescent until they encounter a foreign microorganism or inflammatory stimulus. An immune response to foreign antigen requires the presence of APCs (usually either macrophages or dendritic cells) in combination with B or T cells. Skin-resident APCs exposed to irradiated *S. mansoni* cercariae emigrate from the skin and are able to induce proliferation of *S. mansoni*-specific lymphocytes, and the proliferation of the lymphocytes is dependent on major histocompatibility complex (MHC) II interaction and cell-to-cell contact between APCs and lymphocytes[5]. Different pathogen-associated molecular patterns (PAMPs) may induce different immune responses through different pattern recognition receptors (PRRs). Whether normal cercaria antigen (NCA) and UV-radiation-attenuated cercaria antigen (UVACA) can induce different activation results on antigen presenting cells has not been observed.

Macrophages could be activated by schistosome larvae and produce cytokines, including IL-6, IL-12p40 and IL-10[6]. Macrophage activation was shown to play a role in immunity to schistosome infection[7], and it has been suggested that activated macrophages act as immune effector cells *in vivo*[8]. MHC II plays a critical role in presenting antigen from foreign pathogens and initiating the activation of an antigen specific immune response. IFN-γ was proved to be an important cytokine in activating macrophages in lungs for the immunological killing of *S. mansoni* larvae and playing a critical role in protective immunity against *schistosome* infection[9]. IFN-γ is also well-known for its effect in promoting an immune response, including up-regulating MHC II, especially for macrophages.

To obtain a clear understanding of whether changed innate immune responses might ensue with UV-radiation of cercariae, we compared the immune responses activated with NCA and UVACA, respectively, in the presence of IFN-γ. A mouse macrophage model cell line (RAW 264.7) was used in an attempt to reveal the differences in the responses induced by NCA and UVACA, which might lead to an understanding, at least in part, of the protection induced with UVAC.

## MATERIALS AND METHODS

### Reagents

The rmIFN-γ was purchased from PeproTech, UK. Dulbecco's modified Eagle's medium (DMEM) (Gibco, USA) was supplemented with 4.5 g/L D-glucose, 2 mmol/L L-glutamine, 10% heat-inactivated fetal bovine serum (PAA Laboratories, Austria), 100 U of penicillin and 100 µg of streptomycin per ML (complete DMEM medium) before use. Phycoerythrin (PE)-conjugated rat anti-mouse MHC II (I-A/I-E) (M5/114.15.2) and PE-conjugated rat IgG2a isotype control were obtained from eBioscience, USA. Mouse IL-10 and mouse IL-6 ELISA kits were from Bender MedSystems, Austria. The mouse prostaglandin E_2_ (PGE_2_) EIA Kit was from Cayman Chemical Company, USA. The BCA protein concentration assaying kit was from Pierce Company, Germany.

### Preparation of soluble cercaria antigens

The preparation of soluble cercaria antigens has been described eleswhere[3]. In short, the cercariae of *S. japonicm* were recovered from *Oncomelania* snails infected with *S. japonicum* miracidium in 25°C dechlorinated water. The recovered cercariae were exposed to UV rays at 400 µW/cm^2^ for 1 min (UVAC) or not (NC), respectively and then used to prepare UVACA or NCA. These recovered cercariae were then centrifuged at 10,000 *g* for 20 min at 4°C to precipitate cercariae The cercariae were transformed to schistosomula through mechanical transformation with a 1 ml-syringe by repeated uptake and expulsion 15 times. These mechanically transformed schistosomula were then resuspended in RPMI 1640 at a density of 2,000/mL and cultured at 37°C in a 5% CO_2_ incubator for 3 h. Then the cercariae were collected, recentrifuged, and discarded the supernatants were. The schistosomula were next resuspended in RPMI 1640 at 20,000/mL, followed by being frozen and thawed repeatedly. The cercariae were then fragmented by sonication under a ultrasonic intensity of 50 W/cm^2^ for 10 min and centrifuged at 7,000 *g* for 5 min. The supernatants provided the NCA and UVACA. The protein concentration was determined by the BCA protein concentration assaying kit using the manufacturer's instructions, and the proteins were kept at -20°C prior to use.

### Cell culture

Murine RAW 264.7 macrophage cells were obtained from ATCC (ATCC TIB-71TM, Manassas, USA) and maintained in complete DMEM medium. Cells were adjusted to a concentration of 1×10^6^ cells/mL in the complete medium for use in all of cellular assays. To ascertain the appropriate concentration of rmIFN-γ used for up-regulation of MHC II expression (4 ng/mL selected, data not shown), RAW 264.7 cells were incubated with serially diluted rmIFN-γ for 48 h. To observe the effect of NCA or UVACA on MHC II expression, RAW 264.7 cells were plated in 96-well plates with medium (untreated), NCA(40 µg/mL) or UVACA (40 µg/mL) in the presence or absence of rmIFN-γ (4 ng/mL) 48 h. Cells cultured with rmIFN-γ (4 ng/mL) alone were used as positive controls. To exclude the possible impact of contaminated endotoxin contained in NCA and UVACA, in some experiments, 5 µg/mL of polymyxin B (PMB) was first incubated with NCA or UVACA in the medium for 30 min before the cells were added into the wells. Culture supernatants were collected for detection of cytokines after 48 h.

### Cell surface staining and flow cytometry

RAW 264.7 cells at a concentration of 1×10^6^ cells/mL were incubated in 96-well plates with 40 µg/mL of NCA or UVACA, with or without IFN-γ (4 ng/mL). The cells cultured with IFN-γ alone or medium alone (untreated) were used as controls. The RAW 264.7 cells cultered for 48 h were washed and incubated with PE-MHC II or isotype-matched control antibody for 30 min on ice. The cells were then washed and detected with a FACScan flow cytometer (Becton-Dickinson, USA), and analyzed by CellQuest software (Becton-Dickinson). The results were expressed as mean fluorescence intensities (MFI).

### Evaluation of cytokines and PGE2 production

Cell culture supernatants were assessed for the concentrations of IL-10, IL-6 and PGE_2_ by commercial enzyme-linked immunosorbent assays (ELISA) using paired cytokine-specific mAbs (IL-10 and IL-6) or PGE_2_ EIA kit, respectively according to the manufacturer's instructions.

### Statistical analysis

All data were expressed as mean±SD. Statistical analysis was carried out with SPSS software (Version 11.5, SPSS Inc., USA). The difference between the mean values in the two groups was evaluated by Student's *t*-test. Difference amang groups were analyzed by one way analysis of variance (ANOVA), and post hoc Student Newman Keuls, SNK method was used for multiple comparisons. Statistical significance was set at *P* < 0.05. All the results were based on three batches of repeated experiments.

## RESULTS

### Schistosomula acquired by mechanical transformation

**Fig.1:**
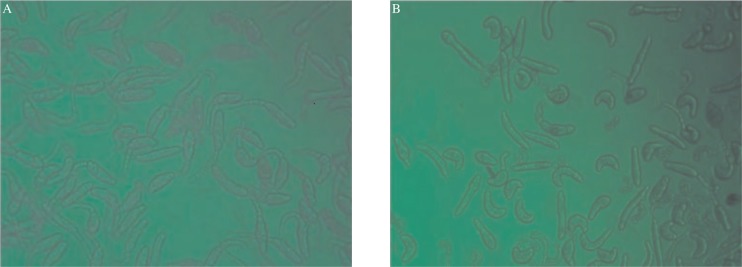
Schistosomula acquired through mechanical transformation(×200). A: *S. japonicum* cercariae were collected and then passed through a 1 mL syringe 15 times following centrifugation and resuspension in medium. B: Most cercariae shed tails and transformed into schistosomula (×200).

Collected cercariae (***Fig. 1A***) were passed through a 1 mL syringe 15 times. When observed under microscope, most cercariae appeared to have shed their tails and had transformed into schistosomula (***Fig. 1B***).

### NCA significantly attenuated IFN-γ-induced MHC II expression on RAW 264.7 cells compared with UVACA

**Fig.2:**
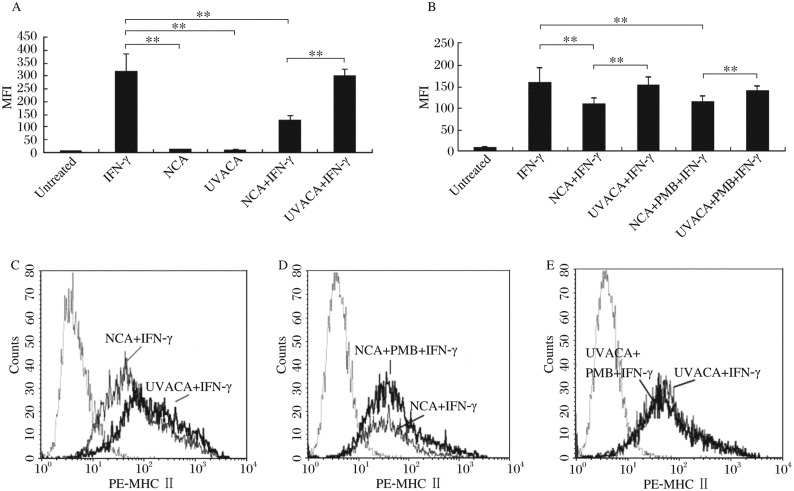
Compared with UVACA, NCA significantly attenuated IFN-γ-induced MHC II expression. A: NCA and UVACA both failed to significantly up-regulate MHC II expression on the surface of RAW 264.7 cells, UVACA had no significant impact on MHC II expression induced by IFN-γ. A and C: In presence of IFN-γ, NCA showed the ability to significantly attenuate IFN-γ-induced MHC II expression compared with UVACA-treated macrophages. B, D and E: Pretreatment with PMB had no effect on the ability of NCA and UVACA to regulate IFN-γ-induced MHC II expression (**P<0.01).

Both NCA and UVACA failed to up-regulate MHC II expression on RAW 264.7 cells (untreated: 7.22±0.43; NCA alone: 11.85±0.35; UVACA alone: 9.68±1.72) (***Fig. 2A***). In the presence of IFN-γ, NCA significantly attenuated IFN-γ-induced MHC II expression, while UVACA had no significant impact on MHC II expression induced by IFN-γ (IFN-γ alone: 317.40±65.10; with UVACA: 301.49±24.31; with NCA: 127.58±16.78) (***Fig. 2A and 2C***). To exclude the possible impact of contaminated endotoxin, we used PMB (5 µg/mL) to neutralize the role of endotoxin. The results showed that, following pretreatment with PMB, NCA still showed the ability to attenuate IFN-γ-induced MHC II expression significantly and UVACA still had no significant impact on IFN-γ-induced MHC II expression (IFN-γ alone: 159.10±33.88; with NCA: 110.03±14.86; with NCA and PMB: 115.40±13.74; with UVACA: 152.32±20.86; with UVACA and PMB: 141.78±8.79) (***Fig. 2B, 2D and 2E***). These data indicated that the difference between NCA and UVACA in regulating IFN-γ-induced MHC II expression was not the result of the possible existence of endotoxin contamination.

### NCA promoted RAW 264.7 cells to increase secretion of IL-6, IL-10 and PGE_2_ significantly in the presence of IFN-γ

**Fig.3:**
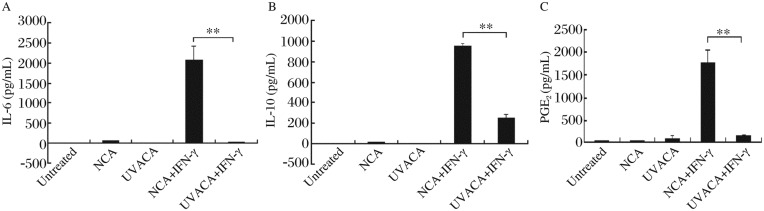
In presence of IFN-γ, compared with UVACA, NCA significantly promoted the production of IL-10, IL-6 and PGE_2_. NCA could promote IL-6 (A) and IL-10 (B) secretion from RAW 264.7 cells compared with untreated and UVACA treated cells, but this effect was not statistically significant. In presence of IFN-γ, NCA significantly promoted IL-6 (A), IL-10 (B) and PGE_2_ (C) production from RAW 264.7 cells compared with UVACA treated cells (**P<0.01).

Macrophages were treated with medium (untreated), NCA (40 µg/mL) or UVACA (40 µg/mL) respectively in the presence or absence of IFN-γ. Forty-eight hours later, culture supernatants were collected for cytokines detection. Compared with untreated cells (IL-6: 0 pg/mL; IL-10: 0 pg/mL) and UVACA-treated cells [IL-6:(1.51±2.61) pg/mL; IL-10:(0.20±0.41) pg/mL], NCA could promote IL-6 and IL-10 secretion by RAW 264.7 cells [IL-6:(63.51±5.58) pg/mL; IL-10:(12.42±2.29) pg/mL], but these differences were not statistically significant (*P* > 0.05) (***Fig. 3A and 3B***). In presence of IFN-γ, secretions of IL-6 (***Fig. 3A***), IL-10 (***Fig. 3B***) and PGE_2_ [untreated:(15.96±2.54) pg/mL; NCA alone:(22.49±1.91) pg/mL; UVACA alone:(105.07±62.76) pg/mL] (***Fig. 3C***) were significantly stimulated from RAW 264.7 cells with NCA [IL-6:(2,075.48±342.43) pg/mL; IL-10:(951.11±24.71)pg/mL; PGE_2_:(1,768.03±279.35) pg/mL] compared with cells stimulated by UVACA [IL-6:(21.56±5.89) pg/mL; IL-10:(256.73±25.74) pg/mL; PGE_2_:(164.47±24.02) pg/mL].

## DISCUSSION

Attenuation with UV light can confer immunizing capacity on cercariae, as shown in *S. mansoni*[10] and *S. japonicum*[11],[12]. The factors related to the induction of protection have been studied in detail on the attenuated -cercaria vaccine model, but the immune mechanisms for the acquired protection remain to be intensively investigated. The expression of the proteins of *S. japonicum* cercariae could be significantly changed by UV-radiation, which was demonstrated by comparative soluble proteomic analysis of NC and UVAC[3]. These changed proteins mainly corresponded to structural and motor proteins such as actin, energy metabolism associated enzymes (*e.g*., glyceraldehydes-3-phosphate dehydrogenase), signaling transduction pathway-associated molecules (*e.g*., 14-3-3 protein), heat shock protein families (*e.g*., HSP 70 family), and other functional proteins (20S proteasome)[3].

Conserved structures on microbes designated as PAMPs might be recognized by PRRs expressed on APCs and lead to an inflammatory response[13]. Thus, recognization of PAMPs by PRRs plays a critical role in the production of protection against invading microorganisms by linking the innate and adaptive parts of the immune system[14]. Whether these changed proteins contained in UVAC can act as PAMPs and induce different immune responses from those induced by NC on APCs has not yet been determined. The data obtained from our study provide the first line of information in this area.

It was reported that macrophages could phagocytize execretory/secretory materials released by cercaria and could reach the sdLNs[15] to play a role in response to invading schistosome cercariae[16]–[18]. Previous studies suggested that DCs were mainly responsible for immune priming, while macrophages were mainly responsible for immune regulation in the development of response to *S. mansoni* cercariae[15]. In our study, in the presence of IFN-γ, NCA promoted macrophages to increase secretion of IL-6, PGE_2_ and IL-10 significantly. Comparatively, in the presence of IFN-γ, UVACA only promoted IL-10 production, and showed no significant impact on the production of IL-6 and PGE_2_. IL-10 and PGE_2_[19] are both well known for their anti-inflammatory effects, and IL-6 is known for both its pro- and anti-inflammatory activities[20]. It has also been demonstrated that IL-6[21], IL-10[22] and PGE_2_[23] induced by some pathogens from macrophages could down-regulate MHC II expression to suppress the immune responses. This is consistent with the fact that MHC II expression induced by IFN-γ could be significantly attenuated by NCA but not by UVACA. In view of the important role of PAMPs-PRRs recognition in initiating downstream signaling and inducing cytokine expression, the differences in cytokine expression between NCA- and UVACA-activated macrophages in the presence of IFN-γ might result from UVACA PAMPs being changed by the UV-radiation. The significant differences in cytokine expression, including IL-6 and IL-10, PGE_2_ and MHC II expression between NCA- and UVACA-activated macrophages also suggested that macrophages might mainly exert their immune regulation role in the development of the response to *schistosome* cercariae, as reported previously[15].

IL-10 has wide-ranging regulatory effects upon antigen presentation, costimulation and the development of acquired T-cell responses[24]. Radiation-attenuated cercariae, which induced high levels of protective immunity in mice, failed to induce IL-10 production[25]. IL-10-deficient mice had an enhanced inflammatory response in the skin[26], which was coincident with elevated expression of pro-inflammatory cytokines and MHC II within the skin[27]. These mice also had enhanced Th1-type responses[28] and a high level of immunity when exposed to multiple doses of radiation-attenuated cercariae[28]. This suggested that IL-10 exerted an anti-inflammatory role and suppressed MHC II expression in NC infection. IL-10 appears to be one of the key regulatory mediators of the immune response in skin. PGE_2_ could promote production of IL-10[29] and has been proposed as the priming mediator of schistosome-induced immunoregulation in the mouse skin[26]. Our results supplied further evidence for the role of IL-10, PGE_2_ and their relationship with MHC II expression regulated by NCA, which is in accordance with the above findings.

IFN-γ is a potent activator for macrophages, including a significant up-regulation of the expression of MHC II molecules on the surface of macrophages. IFN-γ was the major effector in the regulation of immune responses in the animal models infected with *schistosome*. Depletion studies in mice using monoclonal antibodies against IFN-γ[30] or knockout of the IFN-γ receptor geneγ receptor gene[31] showed a significant reduction in vaccine-induced protection against *S. mansoni*. A study on the transcription level of IFN-γ and IL-4 in splenic CD4^+^ T cells revealed that attenuated cercariae elicited predominantly a Th1 response in mice at the early stage of vaccination, whereas normal cercariae stimulated primarily Th2-dependent responses[4]. Analysis of the gene profile of the sdLN cells demonstrated that the level of IFN-γ was significantly higher in vaccinated mice than in those mice infected at days 4, 7 and 14 post-vaccination or post-infection[4]. All these results alerted us to the fact that the regulatory effects of IFN-γ are always associated with the whole immune responses elicited in a schistosome infection, and that they must be considered in any studies of the mechanisms of the immune responses in schistosomiasis, innate or adapted, elicited by the infection.

In conclusion, the significant response differences between NCA- and UVACA-treated macrophages in the presence of IFN-γ, as shown in this paper, supplies important evidence that UV-radiation might subvert the immunogenicity of NC, resulting in an essential change of immune recognition and consequent regulation. Thus the innate immune cells may be able to distinguish between normal and irradiated parasites, raising speculation that irradiation inhibits the activity of other parasite molecules or PAMPs with regulatory activity, although it is still unclear how irradiation can affect the expression of proteins that preform in the cercariae.
